# Integrated Transcriptomic and Un-Targeted Metabolomics Analysis Reveals Mulberry Fruit (*Morus atropurpurea*) in Response to Sclerotiniose Pathogen *Ciboria shiraiana* Infection

**DOI:** 10.3390/ijms21051789

**Published:** 2020-03-05

**Authors:** Lijun Bao, Hongpeng Gao, Zelin Zheng, Xiaoxiao Zhao, Minjuan Zhang, Feng Jiao, Chao Su, Yonghua Qian

**Affiliations:** College of Animal Science and Technology, Northwest A&F University, Yangling 712100, China; baolijun@nwafu.edu.cn (L.B.); gaohp9025@163.com (H.G.); zhengzelin1995@sina.com (Z.Z.); yeyu10963@163.com (X.Z.); mjzhang1008@nwsuaf.edu.cn (M.Z.); fjiao@nwsuaf.edu.cn (F.J.)

**Keywords:** mulberry fruit, sclerotiniose, *Ciboria shiraiana*, transcriptome, metabolite, eugenol/isoeugenol

## Abstract

Mulberry sclerotiniose caused by *Ciboria shiraiana* is a devastating disease of mulberry (*Morus alba* L.) fruit in Northwest China. At present, no disease-resistant varieties are used in production, as the molecular mechanisms of this disease are not well understood. In this study, to explore new prevention methods and provide direction for molecular breeding, transcriptomic sequencing and un-targeted metabolomics were performed on healthy (CK), early-stage diseased (HB1), and middle-stage diseased (HB2) mulberry fruits. Functional annotation, gene ontology, a Kyoto encyclopedia of genes and genomes (KEGG) analysis, and a Mapman analysis of the differentially expressed genes revealed differential regulation of genes related to plant hormone signal transduction, transcription factors, and phenylpropanoid biosynthesis. A correspondence between the transcript pattern and metabolite profile was observed in the phenylpropanoid biosynthesis pathway. It should be noted that the log2 ratio of eugenol (isoeugenol) in HB1 and HB2 are 85 times and 23 times higher than CK, respectively. Our study shows that phenylpropanoid biosynthesis may play an essential role in response to sclerotiniose pathogen infection and eugenol(isoeugenol) enrichment in mulberry fruit, which may provide a novel method for mulberry sclerotiniose control.

## 1. Introduction

Mulberry fruit is a valuable food because of its high nutritional value, pleasant taste, and large amount of biologically active components that might be associated with some potential pharmacological functions that are beneficial for human health [1,2]. In recent years, with both ecological and economic benefits, large-scale mulberry orchards have been set up and the mulberry fruit industry has developed rapidly in China, becoming a model for the diversified development of mulberry in Northwest China. However, incidences of disease in the fruit have also increased. Mulberry sclerotiniose is a devastating fungal disease of mulberry fruit; it not only causes mulberry fruit to lose their color and turn white, but also heavily affects mulberry fruit quality and yield.

Mulberry sclerotiniose can be classified into hypertrophy sorosis sclerotiniose, reduced sorosis sclerotiniose, and small particles sorosis sclerotiniose, according to its morphology and symptoms. Hypertrophy sorosis sclerotiniose is a common and major disease in Northwest China, and its pathogen is *Ciboria shiraiana* or *Sclerotinia sclerotinia*. The diseased fruit fall onto the ground and the pathogen overwinter in the soil as sclerotium. During the mulberry flowering time of the following spring, the sclerotium germinates and the apothecium forms. The ascospore infects the stigma of mulberry pistillate, specifically when the apothecium has matured, and the hyphae multiply, eventually forming sclerotia, and thus completing the infection cycle.

Understanding plant responses to pathogen infection is essential to elucidate the mechanisms of plant–microbe interactions and to develop novel strategies for therapy [3]. Unfortunately, the pathogenesis and molecular mechanism of mulberry sclerotiniose have not been well investigated. Of the two pathogens of hypertrophy sorosis sclerotiniose, *S. sclerotinia* was only discovered recently [4]. At present, few genes, such as *CsCelA* [5] and *CsPG1* [6], have been discovered piecemeal in the study of *C. shiraiana*.

Recently, transcriptome, metabolome, and proteome analyses have been applied as powerful tools to explain the plant–pathogen interactions in many plants, such as *Zymoseptoria tritici* [7], *Fusarium culmorum* [8], *Botrytis cinerea* [9], *Bradyrhizobium diazoefficiens* [10], *Candidatus Liberibacter asiaticus* [11], *Xanthomonas oryzae* [12], and *Cucumber mosaic virus* [13].

With the success of *Morus notabilis* genome sequencing [14], multi-omics technology has also been applied in mulberry sclerotiniose. A transcriptomic and proteomic study of *Ciboria carunculoides*, the pathogen of small particles sorosis sclerotinioseis, which has occurred often in east China, was carried out this year [15,16]. In order to provide a global view of *C. shiraiana* infection regulatory mechanisms in mulberry fruit, in this study, the influence of diseased fruit was investigated using transcriptome and metabolite profiling analysis. The differential gene expression during fungal infection was screened, and changes in selected secondary metabolites were recorded. Our research provides foundational information which may lead to a better understanding of the molecular mechanisms of sclerotiniose in mulberry fruit.

## 2. Results

### 2.1. Symptoms of Infected Mulberry Fruits and Fungal Detection

**Figure 1:**
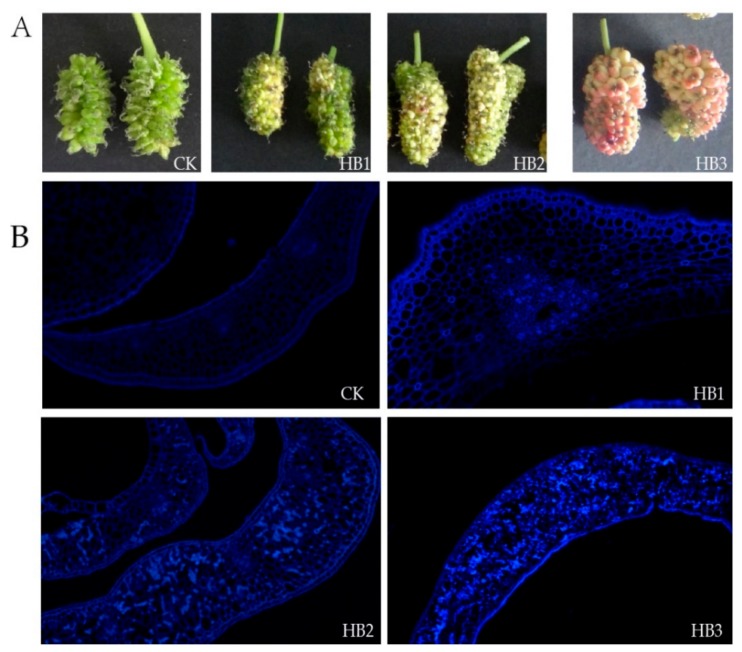
Physical and fluorescent staining of healthy and diseased mulberry fruits infection by *Ciboria shiraiana*. (**A**) Physical appearance of healthy mulberry fruits (CK) and progressive stage of diseased mulberry fruits (HB1, HB2, HB3). (**B**) Fluorescent staining of healthy and diseased mulberry fruits by Calcofluor White Stain.

Healthy mulberry fruits were collected from a normal mulberry orchard and used as the control (CK). Diseased mulberry fruits were divided into three classes: HB1, HB2, and HB3. In the early stage of infection, only a few achenes turned white or gray at stage HB1. The ovary of the mulberry flower swelled due to hyphal proliferation, and the whole fruit was malformed at stage HB2. At stage HB3, the ovary swelled further, some of the diseased fruit turned pink, and the sclerotium was forming (Figure 1A). This was the typical morphological characteristics of hypertrophy sorosis sclerotiniose. In addition, a lot of different levels of diseased mulberry fruits could be found on infected mulberry trees (Figure S1A) and a large number of fungal fruiting bodies were discovered on the ground (Figure S1B,C). Plenty of matured ascospores existed in the apothecium of fruiting bodies (Figure S1D). Moreover, the pathogen in diseased fruits was identified as *C. shiraiana* by fungal ITS primers and a phylogenetic tree analysis, while no pathogens were detected in mulberry fruit from a healthy mulberry orchard (Figure S2A,B). The hyphal of *C. Shiraiana* that had proliferated could be observed by Calcofluor White fluorescent staining (Figure 1B) and pectinase activity increased in diseased fruits (Figure S2C). The plant tissue degraded rapidly, in keeping with hyphal proliferation, and mulberry fruit DNA was barely detected at stage HB3. Therefore, HB1 and HB2 were chosen for subsequent transcriptomic and metabolomic analyses.

### 2.2. Identified Differentially Expressed Genes (DEGs), Their Gene Ontology (GO) Enrichments, and Kyoto Encyclopedia of Genes and Genomes (KEGG) Enrichments by Transcriptome Analysis

**Figure 2:**
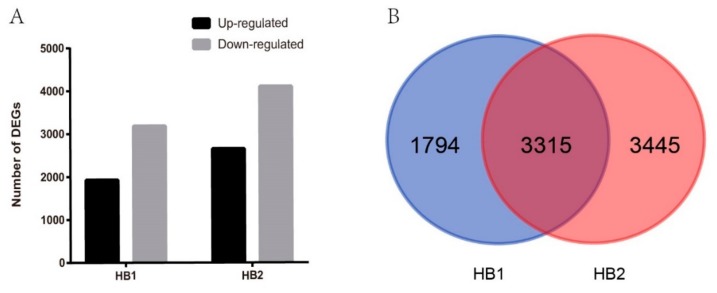
Differential expressed genes (DEGs) in HB1 and HB2 after infection by *Ciboria shiraiana* against CK. (**A**) Up-regulated and down-regulated genes in HB1 and HB2 compared with CK. (**B**) Venn diagrams showing the number of DEGs of infection stage HB1 and HB2.

In order to compare the difference in the transcriptome of mulberry fruit after fungal infection, we performed RNA-seq profiling with the samples CK, HB1, and HB2. A total of 5109 (with 1926 up- and 3183 down-regulated) and 6760 (with 2655 up- and 4105 down-regulated) differentially expressed genes (DEGs) were identified in infection stages HB1 and HB2, respectively, as compared with CK (Figure 2A). As shown in the Venn diagram in Figure 2B, 3315 genes of all DEGs were regulated by *C. shiraiana* at both HB1 and HB2. These results show the differences at the molecular level between healthy fruits and diseased fruits, and also between different disease levels at HB1 and HB2.

**Figure 3:**
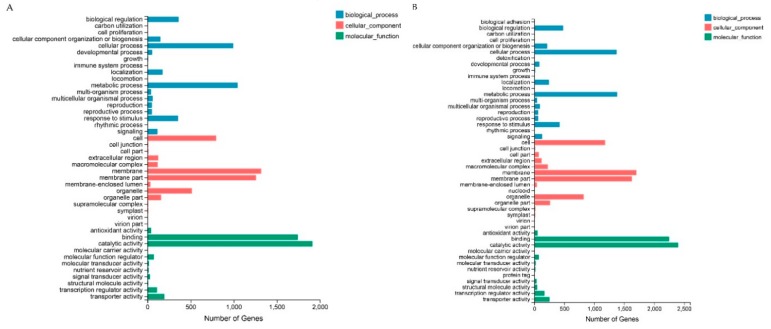
GO analysis of differentially expressed genes (DEGs) in HB1 (**A**) and HB2 (**B**) against CK.

A GO analysis was carried out to classify the functions of the DEGs of HB1 and HB2 compared with healthy mulberry fruit CK. Within the biological process categories, The DEGs from the comparisons of HB1 and HB2 were significantly enriched in the terms of cellular processes, metabolic processes, biological regulation, and response to stimuli. Within the cellular component category, the membrane, membrane parts, cells, and organelles were significantly enriched. Within the molecular function category, the DEGs reveal significant enrichments in categories for binding and catalytic activity. The results indicate that the DEGs were functionally enriched in the same GO terms for the top 10 categories (Figure 3, Table S1). However, further study of GO enrichment showed that the DEGs of HB1 and HB2 were different (Figure S3).

**Figure 4:**
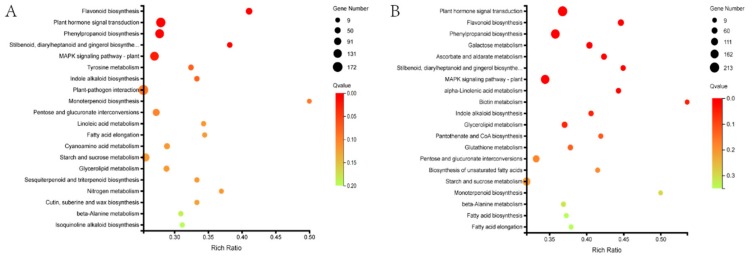
KEGG pathway enrichment analysis of DEGs in HB1 (**A**) andHB2 (**B**).

KEGG pathway analysis of the DEGs identified the terms global and overview maps, carbohydrate metabolism, biosynthesis of other secondary metabolites, signal transduction, amino acid metabolism, and lipid metabolism as being significantly enriched as compared with the whole genomic background in both HB1 and HB2 (Figure S4). KEGG function annotations were used to analyze the KEGG enrichment results as a scatter plot. In both HB1 and HB2, the top three highest significantly enriched KEGG pathways were flavonoid biosynthesis, plant hormone signal transduction, and phenylpropanoid biosynthesis (Figure 4).

To further validate the expression profiles of the RNA-Seq data, eight transcripts from top three of the highest significant terms were selected for analysis using quantification real-time PCR (qRT-PCR). The results from the qRT-PCR analysis are generally in agreement with the expression profiles obtained using the RNA-Seq data (Figure S5, Table S2).

### 2.3. Transcriptomic Response of Mulberry Fruits Infected by C. shiraiana

According to the GO and KEGG enrichment results, plant hormone signal transduction, phenylpropanoid biosynthesis, and transcription factors were analyzed to help understand the response of mulberry fruits to fungal pathogen *C. shiraiana* infection.

#### 2.3.1. Plant Hormone Signal Transduction Pathway

A group of genes changed involved in hormone biosynthesis, mobilization, and signal transduction in response to *C. shiraiana* infection. Transcripts related to plant hormone signal transduction pathway showed that the most were involved in auxin, brassinosteroid, cytokinine, jasmonic acid, gibberellin, abscisic acid, ethylene, salicylic acid, and so on. Log fold ratios for differentially expression genes in HB1 and HB2 compared with CK were shown (Table 1). The transcripts’ up-regulated or down-regulated trends in HB1 and HB2 were similar. Transcripts for abscisic acid receptor (PY1 and PY4), involved in the abscisic acid pathway, were more abundant in HB1 and HB2. Several transcripts related to jasmonic acid and brassinosteroid pathway were down-regulated in HB1 and HB2, including a large number of TIFY protein and protein kinase. Of auxin biosynthesis and signal transduction, transcripts for auxin-responsive protein IAA21 and SAUR36 were down-regulated, while IAA4, IAA29, SAUR24 and SAUR50 were up-regulated. Their detailed roles involved in the responses to *C. shiraiana* infection remain to be explored.

#### 2.3.2. Phenylpropanoid Biosynthesis Pathway

The phenylpropanoid biosynthesis pathway plays an important role in the regulation of secondary metabolites in plants. In this study, 40 DEGs were detected in both HB1 and HB2 (Table 2). Most of the DEGs were up-regulated in HB1 and HB2 compared with CK, such as phenylalanine ammonia-lyase, eugenol synthase, cinnamyl alcohol dehydrogenase, and cinnamoyl-CoA reductase. However, β-glucosidase was down-regulated. It should be noted that both up-regulated and down-regulated genes existed in peroxidase, caffeic acid 3-O-methyltransferase, and 4-coumarate-CoA ligase. 

#### 2.3.3. Transcription Factors

In the present study, 1345 transcription factors (TFs) genes were detected, 306 and 443 TFs were differentially expressed in HB1 and HB2, respectively. There were 122 up-regulated and 184 down-regulated TFs in HB1, and 164 up-regulated and 279 down-regulated TFs in HB2 (Table 3). Of all the differentially expressed TFs, the MYB cluster showed the largest percentage, followed by AP2-EREBP, bHLH, NAC, WRKY, C2H2, MADS, etc. Most of the TFs in HB2, both up- and down- regulated, were increased compared with HB1. Specially, some TFs were down-regulated only in HB2, such as SBP, GRF, C3H, TCP and Tify.

### 2.4. Overview of the Metabolites in the Phenylpropanoid Pathway by Metabolimics Data

In order to obtain an overview of metabolic changes in response to *C. shiraiana* of mulberry fruits, comparative metabolite analyses were performed at CK, HB1, and HB2 using an untargeted metabolomics approach. A principal component analysis (PCA), and a partial least squares discriminant analysis (PLS-DA) were used to analyze these data (Figures S6 and S7). Evaluation parameters of PLS-DA are shown in Table S3. The results show that the three samples were easy to distinguish from the data and the model was stable and reliable.

A total of 1799, 2238, 1642, and 2322 different expression ion numbers were detected from metabolite profiling by negative or positive ionization mode (Table S4). In the phenylpropanoid pathway, 16 metabolites were detected; the *m*/*z* values, retention time, and putative identification of the detected molecular formula are reported in Table 4. The results indicate that phenylalanine, sinapic acid, cinnamaldehyde, *p*-coumaraldehyde, coniferyl acetate, chavicol/isochavicol, methylchavicol/anethole, eugenol/isoeugenol, and methyleugenol/isomethyleugenol were up-regulated, whereas cinnamic acid, *p*-coumaric acid, caffeic acid, caffeyl aldehyde, coniferyl aldehyde, β-d-glucosyl-2-coumarinate, and coumarine were down-regulated in the phenylpropanoid pathway. It should be noted that ratio of eugenol/isoeugenol in HB1 and HB2 were 85-fold and 22-fold higher than that of CK, respectively.

## 3. Discussion

In order to maintain predominance, plants are engaged in a co-evolutionary fight against their pathogens [17]. The widespread changes in plants during pathogen infection make understanding the interactions a huge task [18]. To have a global view of all DEGs in mulberry fruits at HB1 and HB2, a large number of genes involved in metabolism were assessed by a Mapman analysis. Mapman is a common software for displaying large data sets onto pictorial diagrams that symbolically depict areas of biological function [19]. In this study, mainly DEGs of plant hormone signal transduction, phenylpropanoid pathway and transcription factors were discussed. 

**Figure 5:**
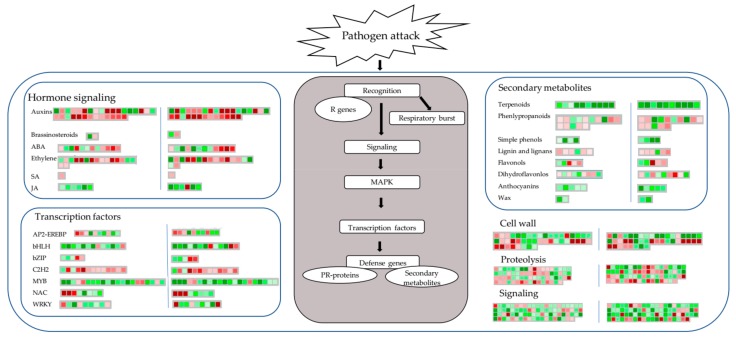
Schematic diagram of biotic stress categories using the Mapman visualization platform. The gene expression ratios of HB1 (left of blue vertical line) and HB2 (right of blue vertical line) compared with CK were used in a Mapman analysis. The gradient of red squares and green squares indicates up- or down-regulated genes involved, respectively.

Figure 5 summarizes the global view of DEGs in response to *C. shiraiana* infection in both HB1 and HB2 using Mapman analysis. Plant hormones play key roles in the regulation of immune responses to microbial pathogens [20,21,22,23]. Jasmonic acid and salicylic acid coordinate growth and defense response upon fungal infection in poplar [24]. Jasmonic acid and terpenoids play roles in resistance against *Phytophthora cinnamomic* in *Zea mays* roots [25]. Ethylene plays a positive role in adapting mulberry to cope with the drought and salinity condition [26]. Transcription factors are also an important factor of gene expression change [27]. *PbMYB10b* and *PbMYB9* regulate anthocyanins and flavonols of the flavonoid biosynthesis in pear fruit [28]. The bHLH factor *VvMYC1* regulates anthocyanin and/or proanthocyanidin (PA) synthesis of the flavonoid pathway in grapevine [29]. In this study, the down-regulation of most MYB and bHLH clusters in HB1 and HB2, which indicates that they may affect the mulberry flavonoid biosynthesis. 

**Figure 6:**
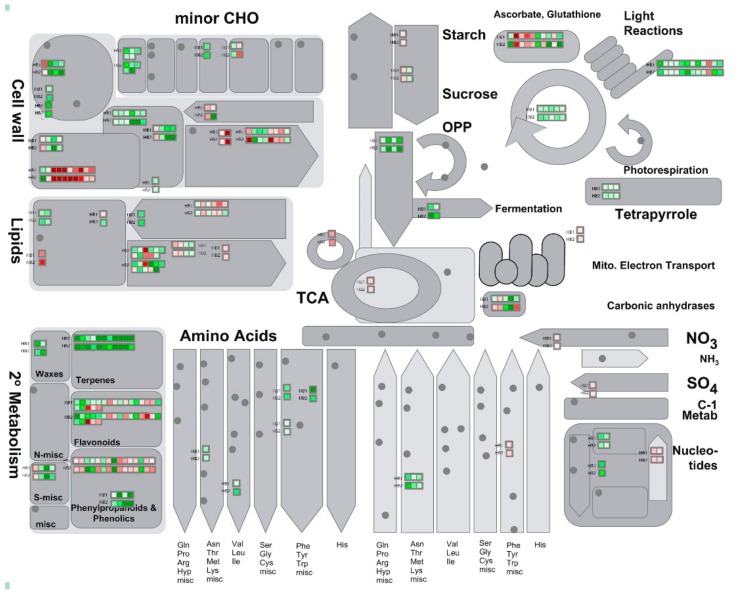
Metabolism overview in Mapman depicting differential regulated genes in HB1 and HB2. Log-fold ratio is shown as a gradient between red (up-regulated) and green (down-regulated).

As shown in Figure 6, in the case of pathogen infection, primary metabolism, such as raffinose synthesis, lipid synthesis, hemicellulose synthesis, cellulose synthesis, and cell wall proteins were decreased, while cell wall modification and degradation, and lipid degradation increased in plants. Light reactions, the Calvin cycle, and glycolysis were also indicated to be suppressed in plants. Changes in plant secondary metabolites were more obvious. Terpenes, phenols, and waxy compounds were suppressed, and phenylpropanoids and flavonoids were increased (Figure 5 and Figure 6). The Mapman analysis results show that most of genes related to secondary metabolism had decreased expression levels, which is similar to the results of the GO and KEGG enrichment analyses. With the infection of pathogens, the infection hyphae rapidly expanded, which took away a lot of plant nutrients, resulting in the inhibition of plant metabolism and the destruction of plant tissue. These also appear during infection by other pathogens. In rice blast disease, the photoassimilates were shifted to mannitol and glycerol for mycelial growth [30]. The bean metabolites that varied in leaves included amines/amino acids, organic acids, phytoalexins, and ureides during *Sclerotinia sclerotiorum* infection [31]. Terpene down-regulation triggers pathogen defense responses in transgenic orange [32]. 

**Figure 7:**
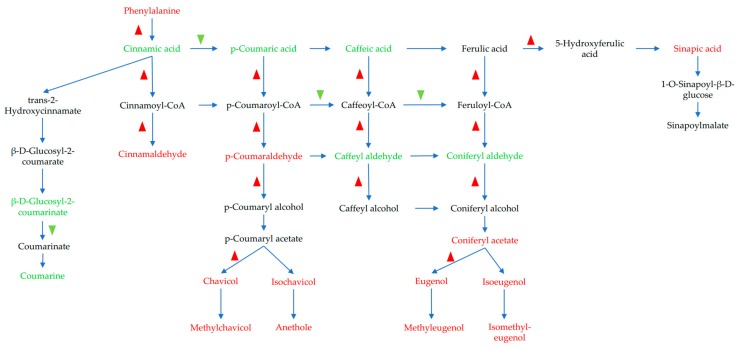
Model summarizing metabolism network in mulberry fruit in response to *C. shiraiana*. The red font indicates significantly up-regulated metabolites (ratio > 1.5); the green font indicates significantly down-regulated metabolites (ratio < 0.5); the black font indicates a low cutoff value (ratio between 0.5 and 1.5) or not detected in this study; the red upward triangle arrow indicates up-regulated genes; the green downward triangle arrow indicates down-regulated genes.

Today, numerous questions about the mechanism of pathogen infection still need to be addressed. Metabolomics is an important platform for studying stress in plant–fungal pathogen interactions [25,33]. We can qualify and quantify the metabolites of plants with biotic stress using modern analytical techniques. When pathogens infect, a large number of plant secondary metabolites are found to be up-regulated. In barley, higher levels of flavonoids, phenylpropanoids, and metabolites of fatty acids and terpenoid pathways were found in the resistant barley lines compared with the susceptible lines during infection with Fusarium [34]. Phenylpropanoid pathway intermediates, like 4-hydroxybenzoate, cinnamic acid, ferulic acid, and caffeic acid, were highly accumulated in the resistant soybean line against *Sclerotinia sclerotiorum* infection [35]. Moreover, in trees displaying symptoms of wood decay, large amounts of lignans were observed [36]. Mulberry trees contain large quantities of flavonoids and other secondary metabolites, such as phenylpropanoids and alkaloids, which have wide roles in defenses against biotic and abiotic stresses [26] and have long been used in Chinese traditional medicine for human disease treatment [37]. A large number of flavonoid metabolites from mulberry fruit have been evaluated both *in vitro* and *in vivo* for their antioxidant activity [38], such as morcin N [39], anthocyanins [40], and so on. Combined with transcriptome and metabolite profiling data, the significant genes and metabolites were found by co-expression analysis of the phenylpropanoid biosynthesis pathway of mulberry fruit in response to *C. shiraiana* (Figure 7, Table S5). We found that a large number of phenylpropanoid pathway intermediates, cinnamic acid, coumaric acid, caffeic acid, caffeyl aldehyde, coniferyl aldehyde were decreased. However, the final metabolites, like chavicol, isochavicol, methychavicol, anethole, eugenol, isoeugenol, methyleugenol, isomethyleugenol, and sinapic acid were increased. In particular, the large increase in eugenol/isoeugenol levels indicates that this compound may play an important role in response to pathogen infection. Eugenol is widely present in plants and is mainly used for antibacterial purposes, and can also be used in various cosmetic flavors and soap flavor formulations. There is no clear evidence that eugenol is carcinogenic. Eugenol exhibits an antimicrobial effect against carbapenem-resistant *Klebsiella pneumoniae* (CRKP) strains and could be potentially used to control CRKP-related infections [41]. The antimicrobial sachet containing the synergistic inhibitory effect of eugenol and citral (SEC) was developed for bread preservation [42]. Eugenol-casein nanoparticles (EC-NPs) exhibited a great antifungal activity against spore germination of fungus and have shown potential as an environmentally friendly preservative in the food industry [43]. Our data reveal important roles of eugenol/isoeugenol enrichment in mulberry fruit in response to *C. shiraiana*, which may provide a novel method for mulberry sclerotiniose control.

## 4. Materials and Methods

### 4.1. Plant Growth Conditions and Fungus Identification

Mulberry cultivar Hongguo2 (*Morus atropurpurea*), which is a widely cultivated mulberry fruit tree in Northwest China, was bred by the Institute of Sericulture and Silk, Northwest A&F University.

A four-year high-yielding fruit mulberry orchard with area of about 3000 m^2^, and a space of 3 m between the rows and 1.5 m between the plants, was located in Guangji Town, Zhouzhi County, Shannxi Province, China. The climate and soil conditions of the orchard were consistent. A total of 20 mulberry trees suffered a serious outbreak of mulberry sclerotiniose on one side of the mulberry orchard near the low slope. A large number of diseased fruits dropped into the ground and formed sclerotia later. About 30 sclerotia per tree were discovered in winter, and PCR amplification was done using ITS sequences as primers, *C. shiraiana* was identified by morphological characters and phylogenetic tree. The diseased fruit could be divided into three stages according to the degree of the disease. Only a few achenes turning light white from green were marked as HB1, and a few appearing white and swelling slightly marked as HB2, while most achenes of mulberry fruit turned white and the whole fruit showed malformation, being marked as HB3. On the other side of mulberry orchard, about 200 m from the diseased area, mulberry fruits grew healthy and no pathogens in the fruit were detected by ITS sequencing, and marked as CK.

HB1, HB2, HB3, CK, fungal fruiting bodies, and sclerotia were collected at the same time. The samples were frozen in liquid nitrogen for storage at −80 °C. Three biological replicates were analyzed using transcriptome sequencing, qRT-PCR validation, and the enzyme activity test. Six biological replicates were used for metabolite profiling. Each replicate consisted of three single fruit, and a single fruit collected from an independent tree.

### 4.2. Pathogen Detection and Enzyme Activity Analysis

Genome DNA of CK, HB1, HB2, and HB3 were extracted from 0.1 g tissue using a rapid plant genomic DNA isolation kit (Sangon, Shanghai, China). Fungal genome DNA were extracted with a rapid fungi genomic DNA isolation kit (Sangon, Shanghai, China), and PCR amplification using fungal universal ITS1 and ITS4 primers.

Pectinase activity was measured by a pectinase assay kit (Jiancheng Biotechnology, Nanjing, China). The activity of the pectinase enzyme was expressed as units per milligrams of protein. Statistical differences were based on one-way ANOVA analysis followed by Tukey’s HSD *t*-test using SPSS 19.

### 4.3. Tissue Paraffin Section and Fluorescent Staning

Several samples were fixed in 4% buffered formalin, embedded in paraffin, and sectioned. For toluidine blue staining, the sections were cut and stained with toluidine blue solution (0.5% toluidine blue, 1% borax). For lactophenol-cotton blue staining, the staining included 0.05 g cotton blue, 20 g phenol crystals, 40 mL glycerol, 20 mL lactic acid, and 20 mL distilled water. For Calcofluor White Staining, one drop of Calcofluor White Stain (18909, Sigma-Aldrich, St. Louis, MO, USA) and one drop of 10% potassium hydroxide were added to the sections, a coverslip was placed over the specimen, let stand for 1 min, and finally examined under UV light (355 nm) [44].

### 4.4. RNA Extraction, RNA Sequencing, and Functional Annotations

Total RNA in each sample was extracted using the MiniBEST Plant RNA Extraction Kit (Takara Bio Inc., Dalian, China) by following the manufacturer’s procedure. An ethanol precipitation protocol and CTAB-PBIOZOL reagent was used for the purification of total RNA. The total RNA quantity and purity were analyzed by a Nano Drop and Agilent 2100 bioanalyzer (Thermo Fisher Scientific, Massachusetts, USA). Oligo (dT)-attached magnetic beads were used to purify mRNA. Sequencing was carried out using the BGISEQ-500 platform (BGI, Shenzhen, China).

The raw reads were filtered by removing adaptor sequences and low-quality sequences which contain more than 5% unknown nucleotides (N), and low-quality reads (those with >20% of bases with a quality value of <15) with SOAPnuke v1.5.2 [45]. The clean reads were aligned to the reference index by HISAT2 v2.0.4 [46]. String Tie (http://ccb.jhu.edu/software/stringtie) v1.0.4 was used to assemble the transcripts depending on the mulberry (*Morus notabilis*) annotation. The gene expression level was calculated with fragments per kilobase of transcript per million (FPKM) mapped reads. FPKM > 1, Fold Change > 2, and Adjusted *p*-value < 0.001 were used as the thresholds for the differential gene expression (DEGs) for the comparisons of HB1 vs. CK and HB2 vs. CK, and enrichment analyses of the gene ontology (GO) and Kyoto Encyclopedia of Genes and Genomes (KEGG) pathways of all the DEGs were analyzed.

### 4.5. Validation of Gene Expression by Quantitative Real-Time PCR (qRT-PCR)

To confirm the RNA-Seq results, 10 genes were selected to examine the consistency of their expression using qRT-PCR assays. All of the gene-specific primers were designed by Primer-BLAST (https://blast.ncbi.nlm.nih.gov/blast.cgi), and the primers are listed in Table S2. PrimeScript™ RT reagent Kit with gDNA Eraser (Perfect Real Time) (TaKaRa, Dalian, China) was used for cDNA synthesis. The ABI 7500 Fast Real-Time PCR Systems (Applied Biosystems, Waltham, Massachusetts, USA) and the abm^®^EvaGreen qPCR MasterMix-No dye kit (Applied Biological Materials Inc., Vancouver, BC, Canada) were used for all of the qRT-PCR assays. The 2^−ΔΔ*C*t^ method was used to calculate the relative gene expression based on the qRT-PCR data [47].

### 4.6. Metabolite Profiling Assay by Ultra-Performance Liquid Chromatography (UPLC) and Mass Spectrometry (MS)

All samples were prepared according to the LC-MS system machine orders. All chromatographic contents were performed using an ultra-performance liquid chromatography (UPLC) system (Waters, Manchester, UK), and ACQUITY UPLC HSS T3 column (100 mm × 2.1 mm, 1.8 μm, Waters, UK) was used for the reversed phase separation. The mobile phase composed of solvent A (water, 0.1% formic acid) and solvent B (acetonitrile, 0.1% formic acid), and the flow rate of the mobile phases was 0.4 mL/min. Gradient elution conditions were set as follows: 0~2 min, 100% A; 2~11 min, 0% to 100% B; 11~13 min, 100% B; 13~15 min, 0% to 100% A. The column oven was maintained at 50 °C and the injection volume for each sample was 10 μL.

A high-resolution tandem mass spectrometer Xevo G2 XS QTOF (Waters, Manchester, UK) was used to test metabolites eluted from the column and operated in both positive and negative ion modes. The capillary and sampling cone voltages were set at 3.0 kV and 40.0 V, respectively, in positive ion mode, while 2.0 kV and 40.0 V were set in negative ion mode. The TOF mass range was between 50 and 1200 Da, and the scan time was set as 0.2 s. For the MS/MS detection, all precursors were fragmented using 20–40 eV, and the scan time was 0.2 s. In the data acquisition process, real-time quality correction was performed on the LE signal every 3 s. At the same time, quality control samples were collected every 10 samples to evaluate the stability of the instrument state during sample collection.

### 4.7. Metabolite Data Processing and Analysis

The raw data, both ESI negative and positive, were pretreated by Progenesis QI v 2.2, (Waters, UK) and metaX [48] (BGI, Shenzhen, China). Principal component analysis (PCA) and partial least square discriminant analysis (PLS-DA) were used for comparing the groups among CK, HB1, and HB2. The metabolites were initially identified based on their retention time, molecular weight, fragmentation patterns, and ultraviolet absorption, compared to standards and published references. The variable importance in the projection (VIP) value and ANOVA methods, under the condition of VIP > 1.0, *p* < 0.05, and fold change < 0.5 or > 1.5, were used for preliminary assessment of significantly different metabolites identified from UPLC–QTOF-MS analysis.

### 4.8. Mapman Analysis

Protein functions were categorized using MapMan software (available online: http://mapman.gabipd.org/). Annotations were transferred to the Arabidopsis genome with consideration for orthologous genes to predict functions of identified proteins from *M. notabilis*.

## Figures and Tables

**Table 1:** Differential expressed genes (DEGs) related to the plant hormone signal transduction pathway. Log-fold ratio is shown as a gradient between red (up-regulated) and green (down-regulated).

Gene ID	Predicted Gene Description	Gene Expression (log_2_ Ratio)	
		HB1	HB2
**Auxin**			
LOC21401961	Auxin-responsive protein SAUR36	−2.51	−1.61
LOC21401600	Probable WRKY transcription factor protein 1	−1.80	−2.52
LOC21388680	Auxin response factor 2B	−1.55	−1.85
LOC21408902	Auxin-responsive protein IAA21	−1.39	−1.45
LOC21386679	Auxin response factor 9	−1.29	−2.27
LOC21403994	Indole-3-acetic acid-amido synthetase GH3.6	−1.27	−1.59
LOC21391765	Auxin-responsive protein SAUR15	−1.08	1.86
LOC21395462	Auxin-induced protein 6B	1.01	1.25
LOC21392112	Auxin-responsive protein SAUR24	1.38	1.60
LOC21402398	Auxin-induced protein AUX22	1.46	1.74
LOC21394746	Probable indole-3-acetic acid-amido synthetase GH3.1	1.51	2.24
LOC21392317	Auxin-responsive protein IAA29	1.66	2.86
LOC21407314	Auxin-responsive protein IAA4	1.74	2.34
LOC21409344	Auxin-responsive protein SAUR50	2.24	3.32
LOC21399379	Auxin-responsive protein IAA4	2.93	4.86
**Cytokinine**			
LOC21385677	Histidine-containing phosphotransfer protein 1	−2.01	−1.49
LOC21407993	Transcription factor BOA	1.15	1.14
LOC21410451	Two-component response regulator ARR5	1.23	0.46
LOC21406708	Two-component response regulator ORR9	1.59	1.27
LOC21406707	Two-component response regulator ARR9	1.61	2.77
LOC21394517	Two-component response regulator ARR17	1.93	2.05
LOC21388228	Two-component response regulator ARR5	2.17	2.89
LOC21384736	Two-component response regulator ARR9	2.43	3.10
**Gibberellin**			
LOC21405471	Transcription factor PIF3	−1.37	−1.51
LOC21398268	Transcription factor PIF1	−1.30	−1.21
LOC21392291	Transcription factor bHLH79	−1.10	−0.94
LOC21402833	Transcription factor bHLH62	1.03	1.16
LOC21399703	Transcription factor BIM1	1.32	0.94
LOC21405713	Transcription factor mef2A	1.41	2.47
LOC21392198	Transcription factor bHLH137	2.48	3.16
LOC21409470	SCARECROW-LIKE protein 7	2.58	2.10
**Abscisic acid**			
LOC21396136	Probable protein phosphatase 2C 75	−2.00	−3.45
LOC21394160	Probable protein phosphatase 2C 6	−1.02	−1.52
LOC21406025	Abscisic acid receptor PYL1	1.01	0.54
LOC21402805	Basic leucine zipper 9	1.36	2.17
LOC21396186	Light-inducible protein CPRF2	2.35	2.54
LOC21390361	Abscisic acid receptor PYL4	2.37	1.69
**Ethylene**			
LOC21398777	Phosphate transporter PHO1 homolog 3	−3.17	−3.55
LOC21393854	Probable serine/threonine-protein kinase DDB_G0271682	−2.79	−2.66
LOC21383879	Serine/threonine-protein kinase CTR1	−1.30	−1.17
LOC21387330	Protein LIGHT-DEPENDENT SHORT HYPOCOTYLS 10	1.16	2.17
LOC21396016	Ethylene-responsive transcription factor ERF096	1.55	2.96
LOC21385827	EIN3-binding F-box protein 1	1.68	1.49
LOC21394948	Phosphate transporter PHO1	1.99	3.13
LOC21408627	Protein LIGHT-DEPENDENT SHORT HYPOCOTYLS 4	2.18	2.73
**Brassinosteroid**			
LOC21404629	Squamosa promoter-binding-like protein 8	−2.52	−4.69
LOC21400451	Teosinte glume architecture 1	−2.48	−1.83
LOC21383985	Probable serine/threonine-protein kinase At4g35230	−1.78	0.19
LOC21397624	Squamosa promoter-binding protein 1	−1.41	−2.00
LOC21396408	Probable disease resistance protein At5g66900	−1.27	−1.67
LOC21407610	Probable serine/threonine-protein kinase At4g35230	−1.24	−1.37
LOC21400300	Probable serine/threonine-protein kinase At5g41260	−1.17	−1.01
LOC21394077	Squamosa promoter-binding-like protein 9	−1.01	−0.88
LOC21393228	LRR receptor-like serine/threonine-protein kinase At2g24230	1.35	0.24
LOC21400957	Tyrosine-sulfated glycopeptide receptor 1	1.58	1.32
LOC21406401	Probable serine/threonine-protein kinase BSK3	1.80	1.87
LOC21405696	Xyloglucan endotransglucosylase/hydrolase protein 23	1.99	2.03
**Jasmonic acid**			
LOC21398450	Transcription factor MYC2	−4.37	−4.89
LOC21386546	Transcription factor bHLH35	−2.63	−2.53
LOC21404490	Protein TIFY 5A	−2.43	−1.15
LOC21386105	Protein TIFY 10A	−2.26	−2.37
LOC21406254	Transcription factor bHLH13	−1.73	−1.88
LOC21387505	Protein TIFY 6B	−1.72	−2.38
LOC21406020	Transcription factor MYC2	−1.46	−1.99
LOC21396351	Protein TIFY 9	−1.42	−1.70
LOC21401260	Transcription factor ILR3	−1.21	−1.50
LOC21384725	Transcription factor bHLH18	1.89	2.90
**Salicylic acid**			
LOC21396732	Pathogenesis-related protein 1	−1.08	−0.76
LOC21403015	Transcription factor TGA1	1.05	1.71
LOC21409923	BTB/POZ domain and ankyrin repeat-containing protein NPR1	1.05	0.69
LOC21387250	Pathogenesis-related protein 1	3.04	2.26

**Table 2:** Classification of the differentially expressed genes identified from HB1 and HB2 compared with CK in the phenylpropanoid biosynthesis pathway. Log-fold ratio is shown as a gradient between red (up-regulated) and green (down-regulated).

Gene ID	Predicted Gene Description	Gene Expression (log_2_ Ratio)	
		HB1	HB2
LOC21407114	Phenylalanine ammonia-lyase	0.52	0.34
LOC21409963	Phenylalanine ammonia-lyase	1.95	1.20
LOC21407113	Phenylalanine ammonia-lyase 1	1.18	0.00
LOC21407115	Phenylalanine ammonia-lyase 1	0.82	0.21
LOC21391685	Eugenol synthase 1	0.91	−0.49
LOC21391683	Eugenol synthase 1	−0.69	−0.49
LOC21391684	Eugenol synthase 1	0.74	0.82
LOC21409656	Cinnamyl alcohol dehydrogenase 1	1.03	0.24
LOC21409658	Cinnamyl alcohol dehydrogenase 1	0.32	0.54
LOC21396683	Cinnamoyl-CoA reductase 1	0.12	−0.65
LOC21398414	Cinnamoyl-CoA reductase 1	1.11	1.43
LOC21398415	Cinnamoyl-CoA reductase 1	0.96	1.39
LOC21398416	Cinnamoyl-CoA reductase 1	0.55	1.04
LOC21398417	Cinnamoyl-CoA reductase 1	0.25	0.36
LOC21399536	Cinnamoyl-CoA reductase 1	0.10	0.54
LOC21401355	β-glucosidase 12	−1.08	−0.69
LOC21385981	β-glucosidase 17	−2.54	−3.96
LOC21402010	β-glucosidase 3	−1.34	−1.58
LOC21385542	Peroxidase 12	−0.63	−1.91
LOC21402709	Peroxidase 17	−1.44	−0.65
LOC21407160	Peroxidase 21	−1.01	−0.26
LOC21387029	Peroxidase 31	1.01	−1.02
LOC21385792	Peroxidase 4	−0.35	0.50
LOC21396696	Peroxidase 42	0.89	0.09
LOC21385871	Peroxidase 45	0.94	0.30
LOC21394066	Peroxidase 55	1.10	1.28
LOC21396430	Peroxidase 72	−1.81	−4.39
LOC21401444	Peroxidase A2	1.59	2.17
LOC21387006	Caffeic acid 3-O-methyltransferase	−0.86	−1.80
LOC21396224	Caffeic acid 3-O-methyltransferase	1.00	2.31
LOC21405422	Caffeic acid 3-O-methyltransferase	0.34	−0.21
LOC21406195	Caffeic acid 3-O-methyltransferase	0.04	0.70
LOC21384962	4-coumarate--CoA ligase 1	−1.06	−2.32
LOC21389140	4-coumarate--CoA ligase 2	0.38	−0.32
LOC21402620	4-coumarate--CoA ligase 2	0.44	0.46
LOC21386277	4-coumarate--CoA ligase-like 7	1.65	0.60
LOC21403500	4-coumarate--CoA ligase-like 7	0.75	0.46
LOC21407615	4-coumarate--CoA ligase-like 5	−1.95	−1.97
LOC21384166	4-coumarate--CoA ligase-like 9	−0.79	−1.34
LOC21409371	Cytochrome P450 84A1	2.11	2.35

**Table 3:** Numbers of up- and down-regulated transcription factors in HB1 and HB2 compared with CK.

Transcription Factors	HB1		HB2	
	Up-Regulated	Down-Regulated	Up-Regulated	Down-Regulated
MYB	17	35	22	45
AP2-EREBP	18	17	23	30
bHLH	11	26	16	25
NAC	12	14	15	14
WRKY	4	11	10	11
C2H2	10	5	12	6
MADS	2	10	5	12
ABI3VP1	4	4	4	10
G2-like	2	3	6	6
SBP	0	11	0	11
GRAS	3	6	4	7
LOB	5	1	5	5
GRF	0	6	0	10
C2C2-Dof	6	0	6	2
HSF	4	2	5	3
Trihelix	1	0	2	6
C3H	0	1	0	8
OFP	4	5	2	5
mTERF	4	2	3	4
zf-HD	2	4	1	6
TCP	0	4	0	7
bZIP	1	1	4	3
ARF	0	2	1	6
Tify	0	5	0	5
C2C2-CO-like	2	0	4	1
C2C2-GATA	0	2	1	4
CPP	1	1	0	4
PLATZ	1	0	2	2
FAR1	0	1	2	2
FHA	0	0	0	4
ARR-B	2	0	1	2
SRS	0	0	2	1
E2F-DP	0	0	0	3
LIM	0	3	0	2
HB	1	0	1	1
C2C2-YABBY	2	0	1	0
BSD	1	0	1	0
DBP	1	0	0	1
Sigma70-like	0	1	1	0
TIG	0	1	0	1
S1Fa-like	0	0	1	0
TAZ	0	0	1	0
BES1	0	0	0	1
GeBP	0	0	0	1
SAP	0	0	0	1
TUB	0	0	0	1
EIL	1	0	0	0

**Table 4:** Annotation of distinguishable metabolites belonging to phenylpropanoid pathway and related to *Ciboria shiraiana*-induced metabolic reprogramming in mulberry fruits. The reported ratio for HB1 and HB2 were obtained from a PLS-DA model of HB1, HB2 vs. CK, respectively.

Metabolites	*m*/*z*	Retention Time (min)	Adduct	Ion Mode	Molecular Formula	HB1		HB2		Regulated
						Ratio	*p*-Value	Ratio	*p*-Value	
Phenylalanine	164.0707	5.026367	M − H	neg	C_9_H_11_NO_2_	2.1720	1.29 × 10^−2^	-	-	up
Cinnamic acid	149.0596	7.963617	M + H	pos	C_9_H_8_O_2_	11.4905	2.32 × 10^−3^	4.6224	1.84 × 10^−3^	down
*p*-Coumaric acid	163.039	4.4665	M − H	neg	C_9_H_8_O_3_	0.3405	1.27 × 10^−2^	0.3196	3.00 × 10^−4^	down
Caffeic acid	179.0339	4.141967	M − H	neg	C_9_H_8_O_4_	0.1961	1.64 × 10^−2^	0.1146	1.76 × 10^−3^	down
Sinapic acid	223.0598	4.5345	M − H	neg	C_11_H_12_O_5_	2.3948	9.14 × 10^−3^	2.9807	1.97 × 10^−3^	up
Cinnamaldehyde	150.0909	1.136233	M + NH_4_	pos	C_9_H_8_O	8.4712	1.47 × 10^−4^	-	-	up
*p*-Coumaraldehyde	149.0596	7.963617	M + H	pos	C_9_H_8_O_2_	11.4905	2.32 × 10^−3^	4.6224	1.84 × 10^−3^	up
Caffeyl aldehyde	147.0439	4.702383	M + H − H_2_O	pos	C_9_H_8_O_3_	0.3493	9.79 × 10^−3^	0.1463	5.00 × 10^−5^	down
Coniferyl aldehyde	179.0694	4.716667	M + H	pos	C_10_H_10_O_3_	0.5148	1.83 × 10^−4^	0.4065	1.91 × 10^−4^	down
β-d-Glucosyl-2-coumarinate	325.092	4.4665	M − H	neg	C_15_H_18_O_8_	0.3771	7.64 × 10^−3^	0.3288	2.31 × 10^−4^	down
Coumarine	147.0438	4.1807	M + H	pos	C_9_H_6_O_2_	0.4280	3.80 × 10^−3^	0.3453	1.02 × 10^−5^	down
Coniferyl acetate	205.086	7.407367	M + H − H_2_O	pos	C_12_H1_4_O_4_	5.0356	1.44 × 10^−3^	4.4807	6.96 × 10^−6^	up
Chavicol; Isochavicol	135.0802	3.767783	M + H	pos	C_9_H_10_O	4.1766	6.69 × 10^−4^	5.2547	1.35 × 10^−6^	up
Methylchavicol; Anethole	131.0853	8.6643	M + H − H_2_O	pos	C_10_H_12_O	3.4420	7.26 × 10^−5^	3.1045	1.28 × 10^−5^	up
Eugenol; Isoeugenol	182.1174	4.759533	M + NH_4_	pos	C_10_H_12_O_2_	85.0298	3.30 × 10^−6^	22.8484	5.22 × 10^−4^	up
Methyleugenol; Isomethyleugenol	177.0911	7.402767	M − H	neg	C_11_H_14_O_2_	9.5141	2.26 × 10^−2^	7.4019	1.73 × 10^−2^	up

## Supplementary Materials

Supplementary materials can be found at https://www.mdpi.com/1422-0067/21/5/1789/s1.
