# Supplementary material for: Integrated Transcriptomic and Un-Targeted Metabolomics Analysis Reveals Mulberry Fruit (Morus atropurpurea) in Response to Sclerotiniose Pathogen Ciboria shiraiana Infection
Source: Int J Mol Sci. 2020 Mar 5;21(5):1789. doi: 10.3390/ijms21051789 (PMC7084804; doi:10.3390/ijms21051789)
Supplement: Supplementary file 1 [file ijms-21-01789-s001.zip › Figure S1-S7.pdf]

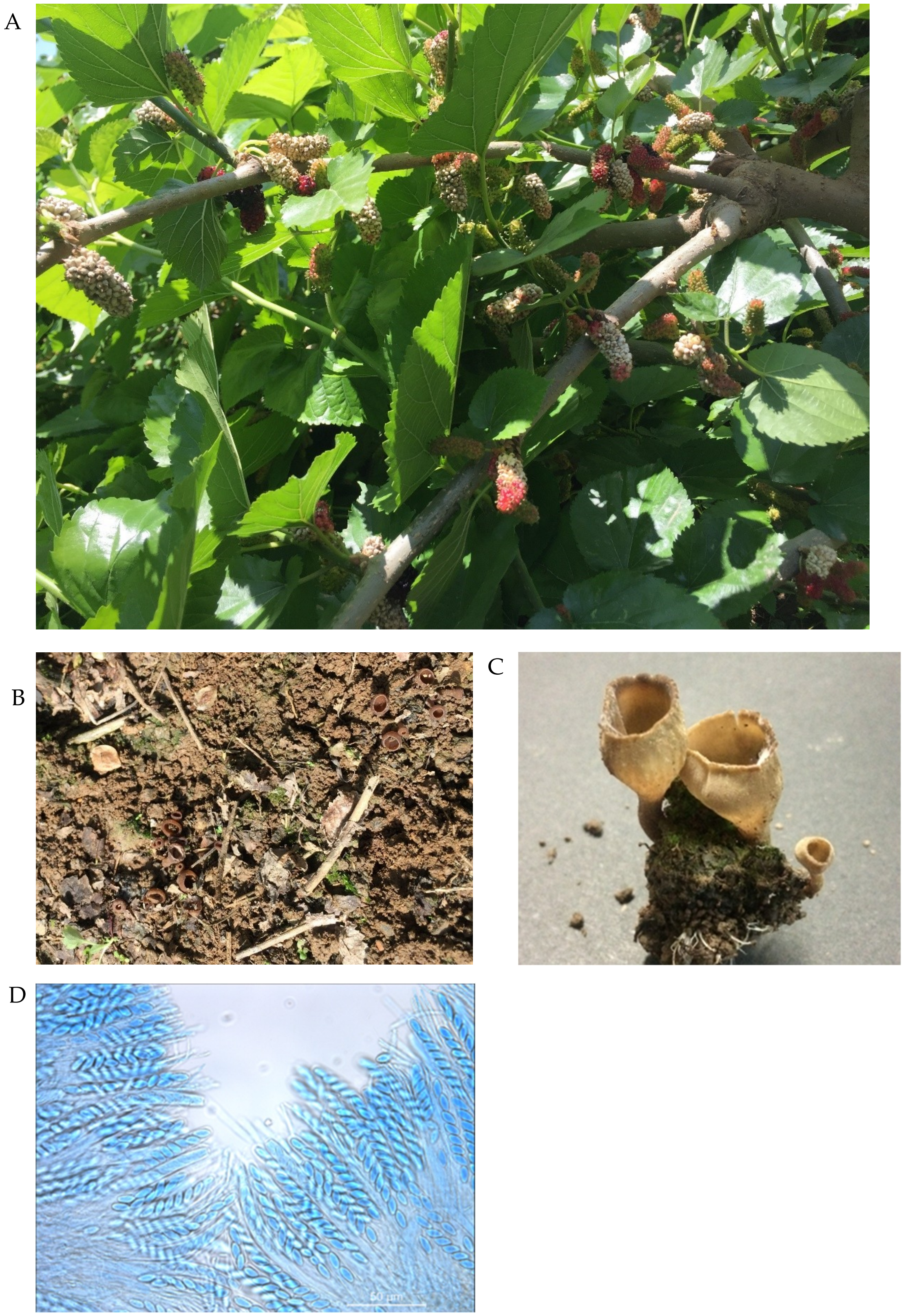

Figure S1. The diseased mulberry fruits and its pathogen. (A) Different levels of diseased mulberry fruits after pathogen infection. (B) Fungal fruiting bodies appeared on the ground. (C) Serval fungal fruiting bodies formed on a single sclerotium. (D) Ascomycetes and ascospores stained with toluidine blue.

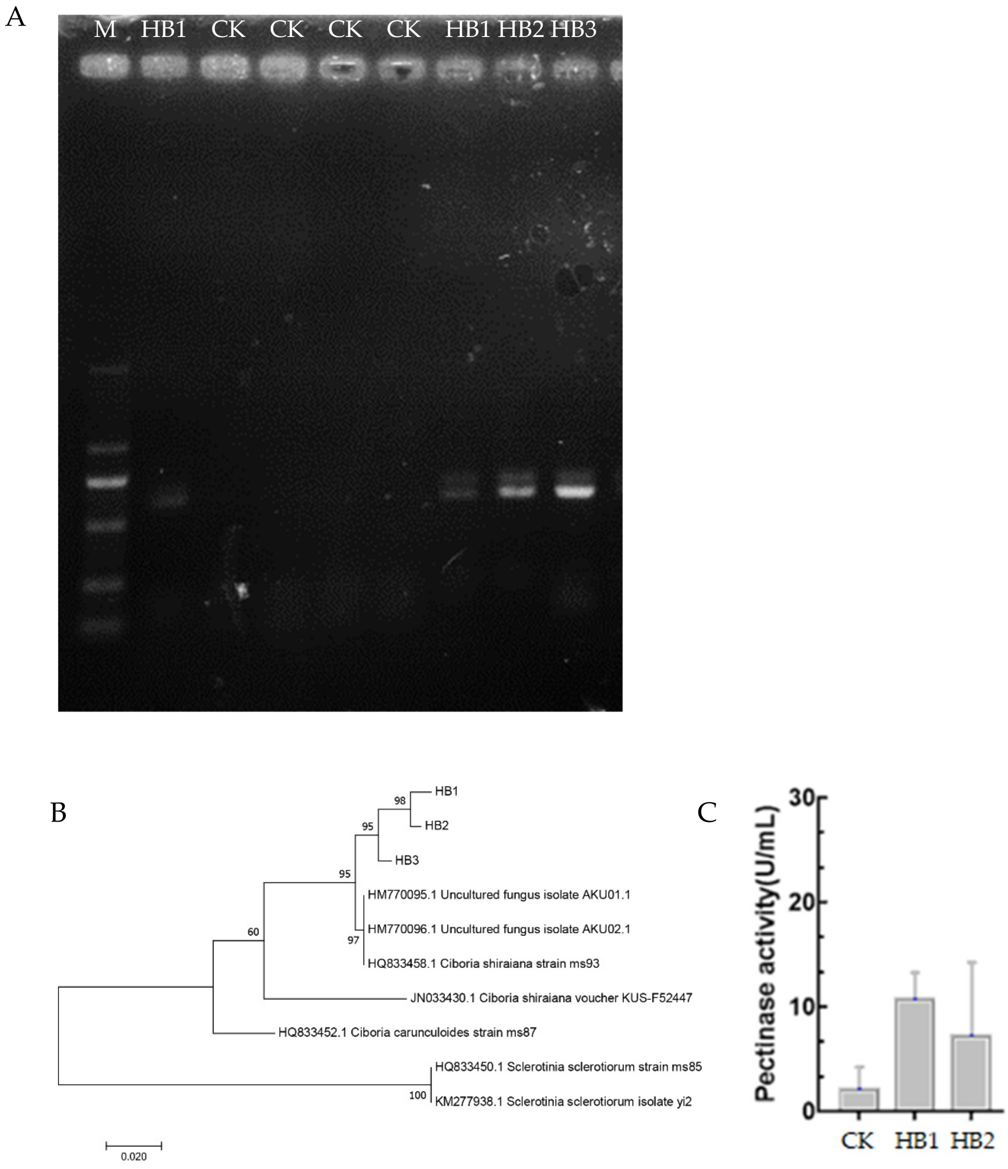

Figure S2. Molecular identification and enzyme activity of pathogen in healthy and diseased fruits. (A) Agarose gel electrophoresis of healthy and diseased fruits tissue, using fungal ITS1 and ITS4 sequences as primers. (B) The phylogenetic tree analysis of HB1, HB2 and HB3. (C) The pectinase activity of healthy and diseased mulberry fruits.

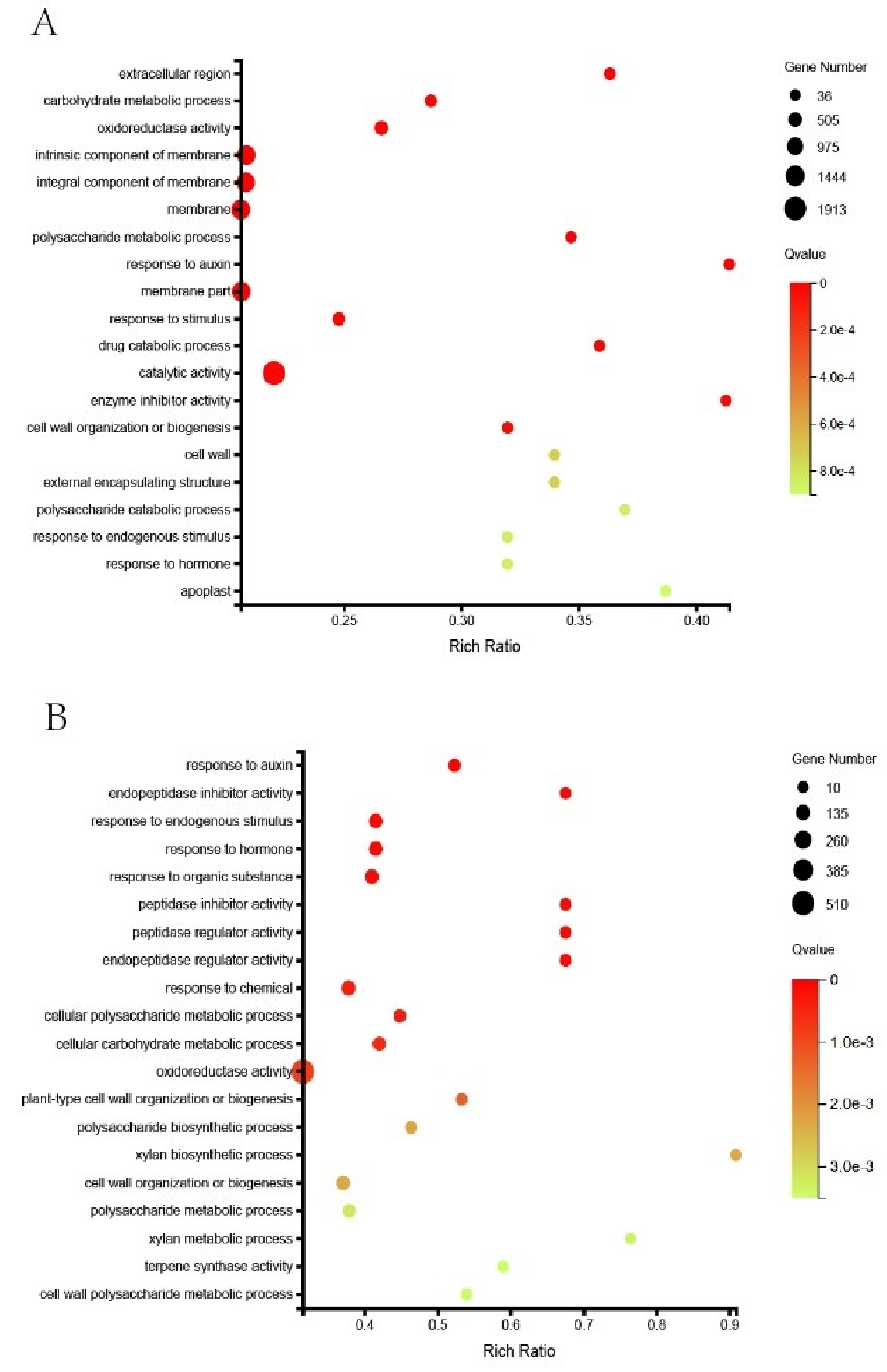

Figure S3. GO enrichment analysis of the DEGs of HB1 (A) and HB2 (B).

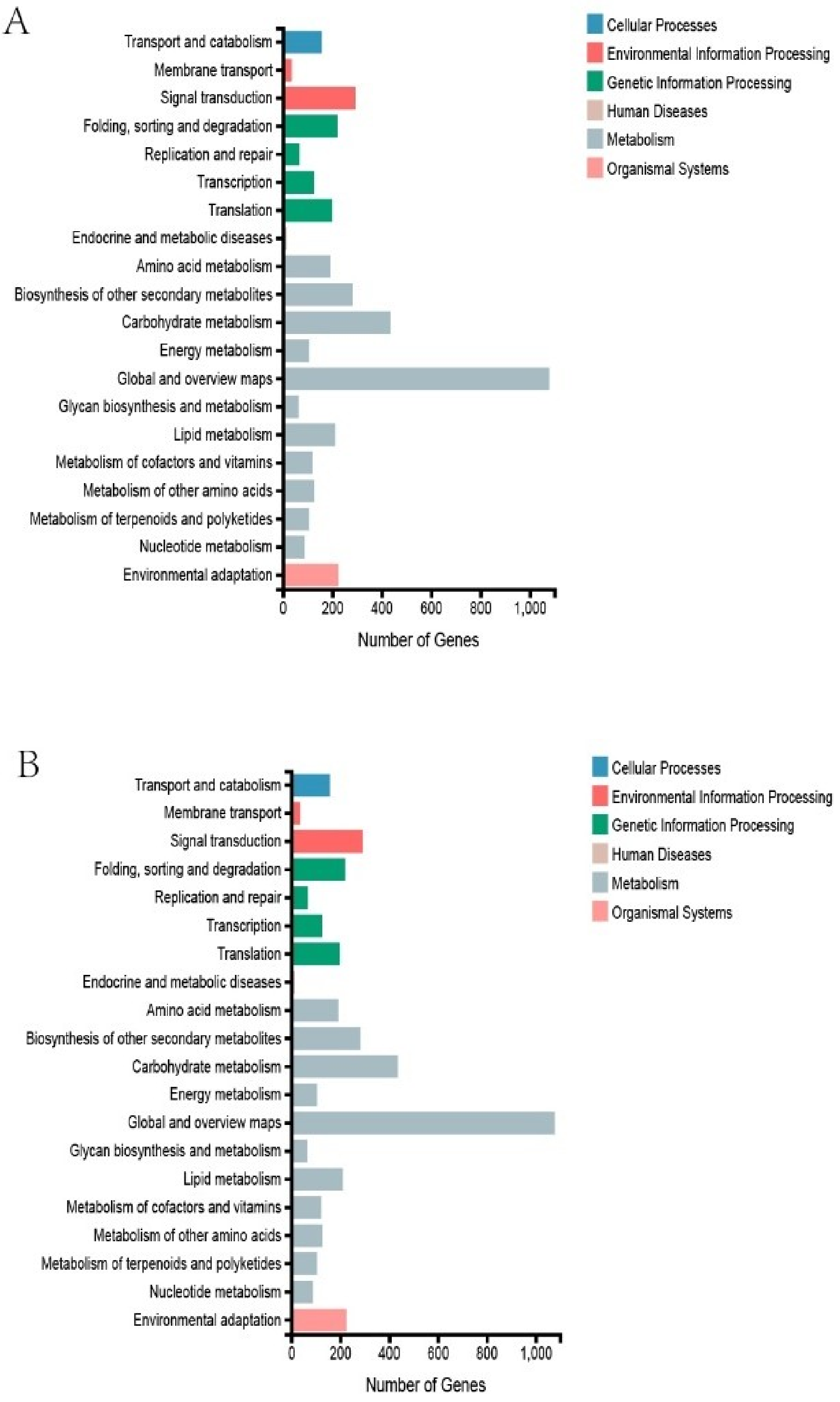

Figure S4. Functional classifications of DEGs in HB1 (A) and HB2 (B) based on KEGG pathways. The X-axis indicates the number of DEGs involved in these pathways. The Y-axis indicates the different terms.

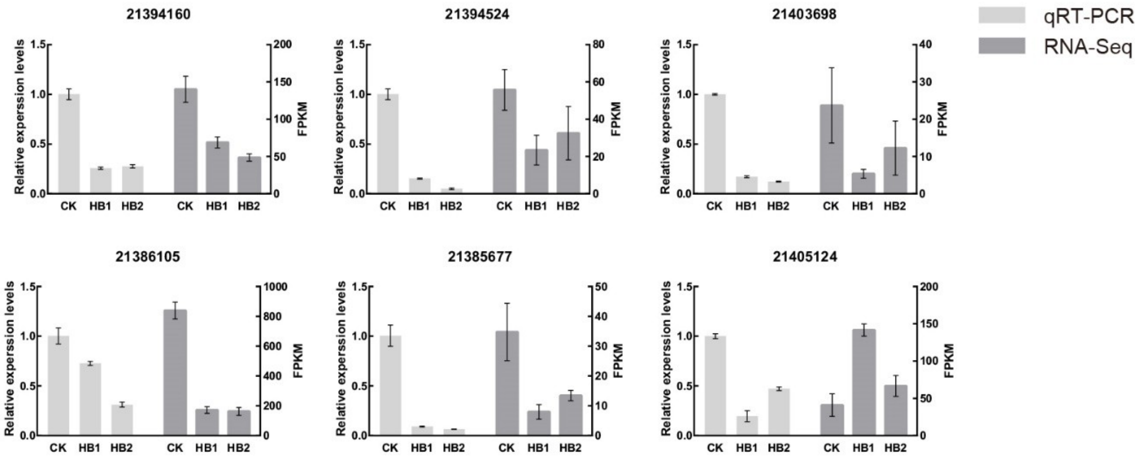

Figure S5. The qRT-PCR validation of transcriptome data.

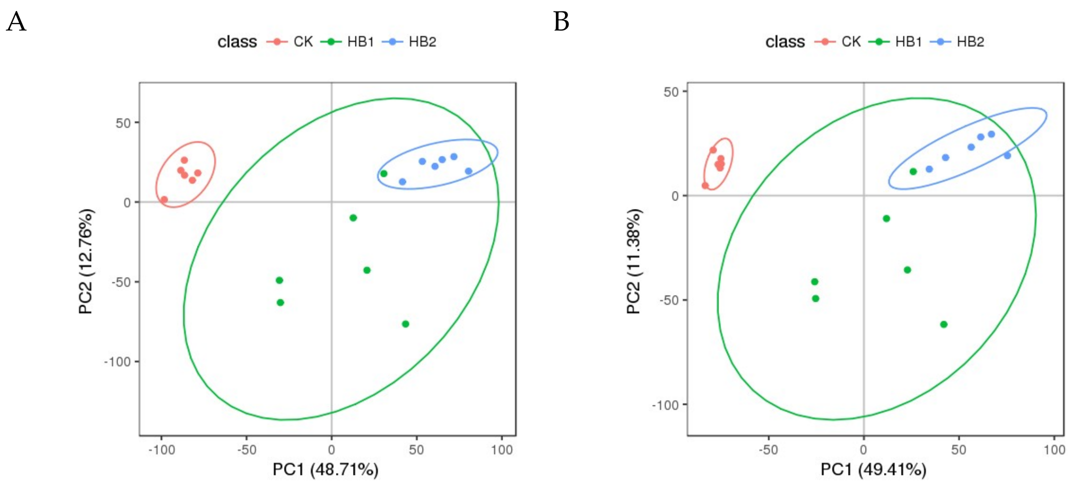

Figure S6. Principal component analysis (PCA) of negative (A) and positive (B) data of CK, HB1, and HB2.

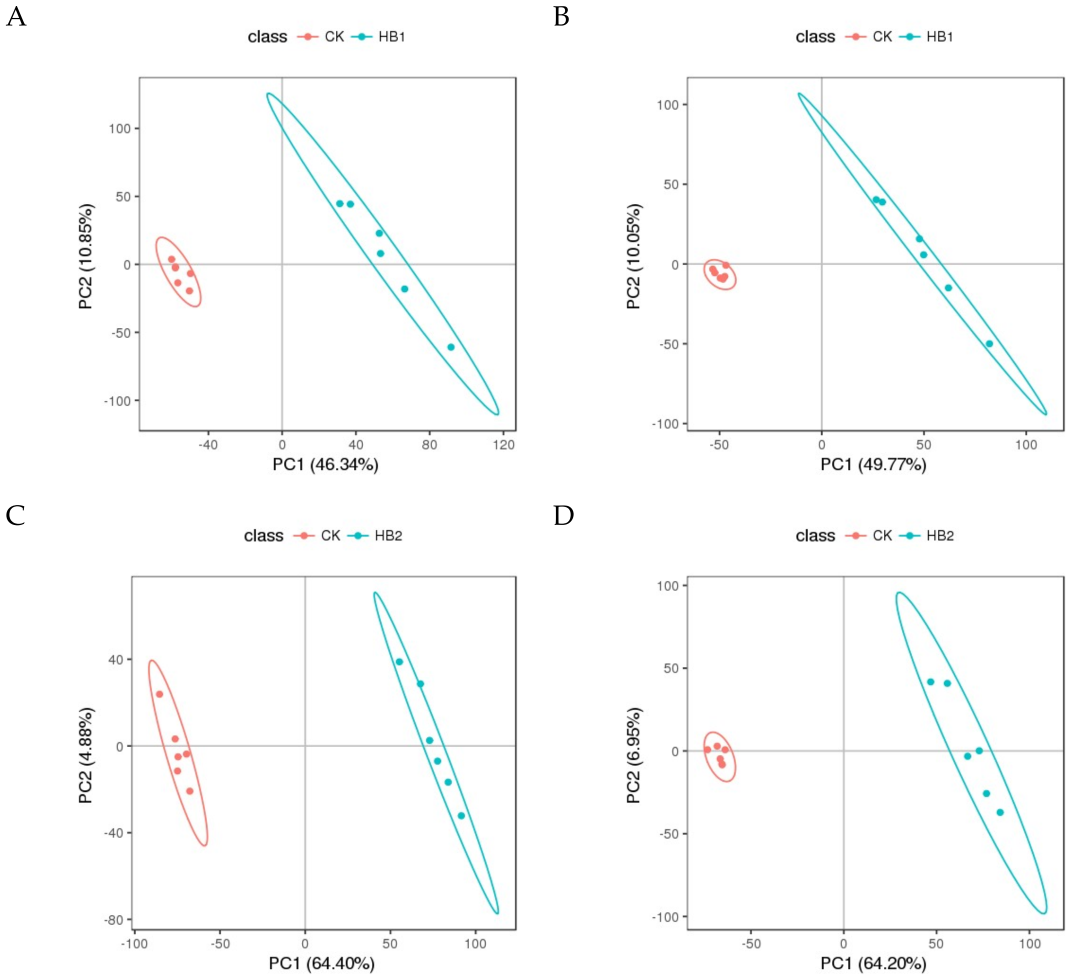

Figure S7. Partial least squares discriminant analysis (PLS-DA) of HB1 and HB2 in negative (A,C) and positive (B,D) model, respectively.
